# Band Engineering and Morphology Control of Oxygen-Incorporated Graphitic Carbon Nitride Porous Nanosheets for Highly Efficient Photocatalytic Hydrogen Evolution

**DOI:** 10.1007/s40820-020-00571-6

**Published:** 2021-01-04

**Authors:** Yunyan Wu, Pan Xiong, Jianchun Wu, Zengliang Huang, Jingwen Sun, Qinqin Liu, Xiaonong Cheng, Juan Yang, Junwu Zhu, Yazhou Zhou

**Affiliations:** 1 Key Laboratory for Soft Chemistry and Functional Materials, Ministry of Education, Nanjing University of Science and Technology, Nanjing, 210094 People’s Republic of China; 2 School of Materials Science and Engineering, Jiangsu University, Zhenjiang, 212013 People’s Republic of China; 3 Institute of Nuclear Science and Technology, Sichuan University, Chengdu, 610064 People’s Republic of China

**Keywords:** Graphitic carbon nitride nanosheet, Hollow morphology, Oxygen incorporating, Multiple thermal treatment, Photocatalytic hydrogen evolution

## Abstract

Graphitic carbon nitride (g-C_3_N_4_)-based photocatalysts have shown great potential in the splitting of water. However, the intrinsic drawbacks of g-C_3_N_4_, such as low surface area, poor diffusion, and charge separation efficiency, remain as the bottleneck to achieve highly efficient hydrogen evolution. Here, a hollow oxygen-incorporated g-C_3_N_4_ nanosheet (OCN) with an improved surface area of 148.5 m^2^ g^−1^ is fabricated by the multiple thermal treatments under the N_2_/O_2_ atmosphere, wherein the C–O bonds are formed through two ways of physical adsorption and doping. The physical characterization and theoretical calculation indicate that the O-adsorption can promote the generation of defects, leading to the formation of hollow morphology, while the O-doping results in reduced band gap of g-C_3_N_4_. The optimized OCN shows an excellent photocatalytic hydrogen evolution activity of 3519.6 μmol g^−1^ h^−1^ for ~ 20 h, which is over four times higher than that of g-C_3_N_4_ (850.1 μmol g^−1^ h^−1^) and outperforms most of the reported g-C_3_N_4_ catalysts.

**Supporting information:**

The online version of this article (10.1007/s40820-020-00571-6) contains supplementary material, which is available to authorized users.

## Highlights

- The multiple thermal treatments strategy is proposed for preparing the hollow oxygen-incorporated g-C_3_N_4_ nanosheets (OCN).
- Oxygen-adsorption creates a lot of defects to the formation of hollow and monolayered structure, while oxygen-doping reduces the band gap significantly.
- OCN exhibits stable and high photocatalytic hydrogen evolution with increased surface area, enhanced charge transport, and reduced band gap.

**Supporting information:**

The online version of this article (10.1007/s40820-020-00571-6) contains supplementary material, which is available to authorized users.

## Graphical abstract

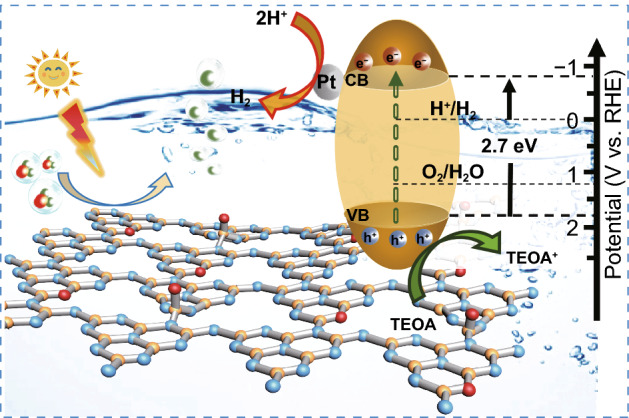

## Introduction

Due to energy and environmental issues, photocatalysis has attracted intensive interests, as it provides a green and potential route for the wide applications in environmental remediation, energy production, and chemical synthesis [1, 2]. During the past several years, various inorganic semiconductors have been developed as photocatalysts for the splitting of water into hydrogen gas under visible light [3, 4]. Graphite-like carbon nitride (g-C_3_N_4_) is not only one of the superior photocatalysts, but also can be used to construct excellent catalysts as substrates owing to high chemical and thermal stability, simple synthesis process, visible-light response, as well as environmentally friendly [5–7]. However, the quality of g-C_3_N_4_ including the thickness, surface area, electronic structure, etc., still needs to be significantly improved to meet the requirement of applications [8, 9] and the morphology design and heteroatom-doping are typical approaches [10–12].

Researches have shown that heteroatom such as nitrogen (N), oxygen (O), sulfur (S), phosphorus (P) dopants play a vital role in promoting the photocatalytic activity of g-C_3_N_4_ through broader light-responsive range, higher light utilization efficiency owing to the reduced band gap [13, 14]. For instance, Li et al. [15] found that P-doping could significantly improve the electronic conductivity of g-C_3_N_4_, leading to the inhibition of recovering of photo-separated charges and holes under visible-light irradiation. Liu et al. [16] proved that the S dopants could donate valence electrons to covalent C atoms, resulting in the narrow band gap and improved photo-reactivity of g-C_3_N_4_. Unfortunately, it has been convinced that these heteroatom dopants can be easily removed, leading to the poor stability of photocatalytic performance [17, 18]. O, a typical abundant element, has been used to improve the photocatalytic performance of g-C_3_N_4_ through the modification of electronic structures and morphology of g-C_3_N_4_. For example, Rodrigues et al. prepared O–g-C_3_N_4_ monolayers by the pyrolysis of melamine under the air atmosphere [19]. Niu et al. [20] utilized a 5 min thermal treatment under the well-ventilated air space to prepare porous O–g-C_3_N_4_. Compared with bulk g-C_3_N_4_, the photocatalytic performance of O–g-C_3_N_4_ was enhanced due to the change in band gaps and improved surface areas [21]. Therefore, O-modified g-C_3_N_4_ is a promising approach to further improve the quality of g-C_3_N_4_.

In this paper, we demonstrate a novel approach to prepare the hollow O-incorporated g-C_3_N_4_ nanosheets (OCN) using the multiple thermal treatments under the N_2_/O_2_ atmosphere. After repeating thermal treatments for three times, the OCN monolayers with uniform pores (~ 25 nm) can be obtained. The surface area was increased to (~ 148.5 m^2^ g^−1^), which is four times higher than that of bulk g-C_3_N_4_ (~ 23.8 m^2^ g^−1^). The obtained OCN exhibited an excellent photocatalytic performance toward hydrogen evolution reaction including a hydrogen evolution rate of 3519.6 μmol g^−1^ h^−1^ for ~ 20 h and quantum efficiency (QE) of 26.96% at 400 nm, outperforming the bulk g-C_3_N_4_ and most of the reported heteroatom-doping g-C_3_N_4_ [22–27]. The physical characterizations and theoretical calculations indicated that the O-adsorption contributed to form a hollow structure by defect creation and the O-doping played a crucial role in the reduction of the band gap of g-C_3_N_4_, leading to the improvement of photocatalytic activity.

## Experimental Section

### Synthesis of OCN

The OCN was prepared by the multiple thermal treatments. In detail, a certain amount of urea powder was placed in a porcelain crucible with a lid. The crucible containing urea was heated in the tube furnace at a temperature of 550 °C for 240 min with a heating rate of 2 °C min^−1^ under the N_2_/O_2_ mixed gas (V/V: 4:1, 50 mL min^−1^). The yellow powder was obtained. Then, the yellow powder was retreated for 60 min with the same condition. The as-prepared product was labeled as OCN-*N* (*N*: the thermal treatment times). Finally, a series of OCN-*N* catalysts, namely OCN-1, OCN-2, OCN-3, and OCN-4, were obtained. The pure bulk g-C_3_N_4_ was prepared with the same procedures of OCN-1 but using melamine under argon atmosphere, which was labeled as MCN.

### Material Characterizations

The crystal structure of products was characterized by X-ray powder diffraction (XRD) on Rigaku/MiniFlex600 powder X-ray with high-intensity Cu Kα. The surface functional groups and chemical compositions of products were performed by KBr pellet pressing method on Fourier transform infrared spectroscopy (FT-IR, Nicolet Avatar-330) and X-ray photoelectron spectroscopy (XPS) on ESCALAB250Xi. Al X-ray was used as the excitation source. The morphological structures of the product were measured by the scanning electron microscope (SEM, Hitachi S4800), transmission electron microscope (TEM, JEOL JEM-2100) operated at 200 kV (Cs 0.5 mm, point resolution 1.9 Å) and atomic force microscopy (AFM, Bruker Multimode 8, Germany). The specific surface areas of samples were measured by N_2_ adsorption–desorption isotherms on a NOVA300e adsorption instrument at 77 K. Then, the corresponding pore-size distributions were calculated using Barrett–Joyner–Halenda (BJH) method. The optical properties of products were tested by UV–vis diffuse-reflectance spectroscopy (UV-2550). Photoluminescence (PL) spectra were measured on a QuantaMaster™ 40 fluorescence spectrophotometer with an incident light of 325 nm. The VB-XPS spectrum was carried out to determine the valence band (VB), then the CB potentials of different photocatalysts were calculated according to the following equations:$E_{{{\text{CB}}}} = E_{{{\text{VB}}}} - E_{{\text{g}}}$, where *E*_CB_, *E*_VB_, and *E*_g_ were the conduction band potential, valence band potential (*E*_VB_ from VB-XPS), band gap (*E*_g_ from UV–vis DRS). Flat band potentials of different samples are obtained by intercepting the tangent of the Mott–Schottky curves. Reactive oxygen species (ROS) including e^−^ and h^+^ from composites solution were identified and quantified with electron spin resonance spectroscopy (ESR, JESFA200).

### Photocatalytic Activity

The photocatalytic hydrogen production reaction was measured in an online photocatalytic hydrogen production system (LbSolar-3AG, PerfectLight, Beijing). The photocatalysts (10 mg) were added into 100 mL aqueous solution containing 90 mL of water and 10 mL of triethanolamine (TEOA) [28–30]. Pt (acting as a co-catalyst) was then deposited onto catalysts by in situ photodeposition method [31]. A 3 wt% (respect to Pt) H_2_PtCl_6_·6H_2_O solution was added and degassed, and then irradiated by 300 W Xenon lamp (PLS-SXE 300C (BF), PerfectLight, Beijing) with an optical filter (*λ* > 420 nm). Gas concentration analysis was performed by using an online gas chromatograph (GC D7900P, TCD detector). Apparent quantum efficiency (AQE) was measured under the same photocatalytic reaction conditions with irradiation light through a cutoff filter (400, 420, 460, or 550 nm) [32, 33]. The AQE was calculated in the Supporting Information (Table S1).

### Photoelectrochemical Experiments

Electrochemical impedance spectroscopy (EIS), transient photocurrent, and Mott–Schottky plots were tested on CHI 660E (Chenhua Instrument, Shanghai, China) with a typical three-electrode cell. The electrode contains a working electrode (prepared sample), a counter electrode (platinum foil), and a reference electrode (Ag/AgCl). Here, 0.2 M sodium sulfate (Na_2_SO_4_) aqueous solution (pH =  ~ 5.8) was employed as the electrolyte solution, and a 300 W Xe lamp equipped with a 420 nm cutoff filter was utilized as the visible-light source. The working electrode was prepared according to the following procedure: 5 mg of the as-prepared photocatalyst was dispersed into a mixed solution containing ethanol (250 μL), ethylene glycol (250 μL), and Nafion (40 μL). The above solution (80 μL) was then dropped onto a precleaned fluorine tin oxide (FTO) glass with an exposed area of 1 cm^2^. The photocurrent responses of the photocatalysts to light switching on and off were measured with 1.2 V bias voltage. EIS spectra were recorded in the range from 0.01 to 10^5^ Hz at an ac voltage of 10 mV. Mott–Schottky plots of material were then tested at 500 Hz frequencies by using the impedance-potential mode.

### Theoretical Calculation

The spin-polarized density functional theory (DFT) calculations were carried out by using the Vienna Ab-initio simulation package (VASP) [34]. All the calculations were performed to describe the electron–ion interaction by using a plane-wave basis and a projector augmented wave (PAW) method. The generalized-gradient approximations (GGA) with the standard norm conserving Perdew–Burke–Ernzerhof (PBE) and Heyd–Scuseria–Ernzerhof (HSE06) exchange–correlation functionals were employed to obtain the exchange and correlation energy [35]. The energy cutoff for the plane-wave basis wave functions was 400 eV and the Gaussian smearing width was set as 0.05 eV. The Brillouin zone was sampled by a Gamma centered 3 × 3 × 1 Monkhorst Pack grid. All atoms were converged to 0.01 eV Å^−1^. A 3 × 3 × 1 supercell model of monolayer g-C_3_N_4_ was first relaxed by PBE approximations, and then the HSE06 calculations were employed to describe electronic structures of different g-C_3_N_4_ samples. Compared with conventional DFT, our calculations could obtain the more exact electronic structures and band gaps by means of HSE06 hybrid density functionals. The calculated N–C bond length is consistent with the published values [35, 36]. The energy balance (Eb) is defined as:${\text{Eb}} = E({\text{O}}{-}{\text{g - C}}_{{3}} {\text{N}}_{{4}} ) - (E({\text{g - C}}_{{3}} {\text{N}}_{{4}} ) + E({\text{O}}) - nE({\text{N}}))$, n = 0 (O-adsorption) or 1 (O-doping), where *E*(O) and *E*(N) refer to the total energy of O and N species, which is calculated from the isolated O_2_ and N_2_ molecule [21, 37].

## Results and Discussion

**Fig. 1:**
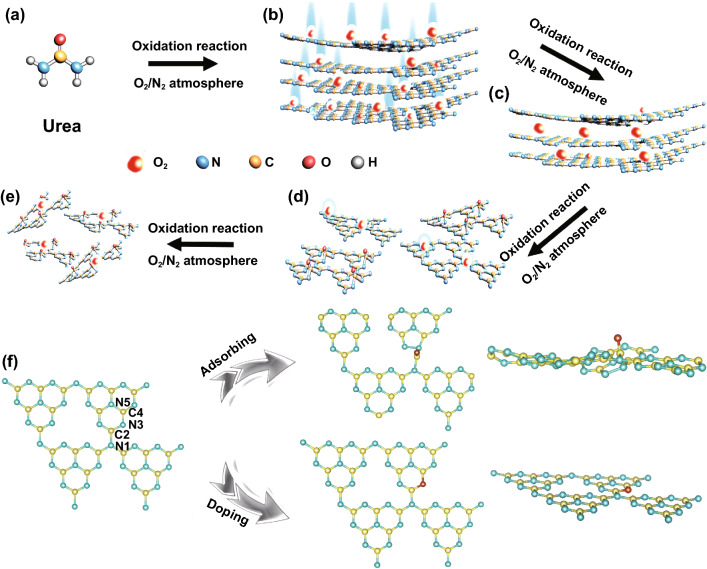
**a**–**e** Synthetic route and **f** DFT computational simulation of OCN which oxygen atoms locate at C_2_ (adsorbing site) and N_3_ (doping site) with low energy

The OCN material was prepared by the multiple thermal treatments, and the critical concept for synthesis is illustrated in Fig. 1. Under the first thermal treatment by urea in the N_2_/O_2_ atmosphere, O atoms adsorbed and combined with g-C_3_N_4_ (Fig. 1a, b). When the OCN-1 was retreated under the N_2_/O_2_ atmosphere, O atoms were easy to be doped into the skeleton (Fig. 1c, d). Using the multiple thermal treatments, a series of oxygen-incorporated sheets OCN-2 and OCN-3 can be obtained and tuned. However, excessive treatments may lead to the fragmentation of OCN-4 material. In order to further confirm the exact position of O atom in OCN, the DFT calculations were employed. The five possible action sites (N_1_, C_2_, N_3,_ C_4_, N_5_) were proposed based on the locations of oxygen atoms (Fig. S1). The HSE06 calculated results showed that the O structure with C_2_ site has a lowest adsorbing energy (-2.17 eV), while the structure with N_3_ site has a lowest doping energy (− 1.63 eV). Thus, it is likely that both O-adsorption and O-doping in g-C_3_N_4_ skeleton were existed to construct the C–O bond in OCN during the multiple thermal treatments (Fig. 1f). Different from O-doping, O-adsorption can make g-C_3_N_4_ distorted.

**Fig. 2:**
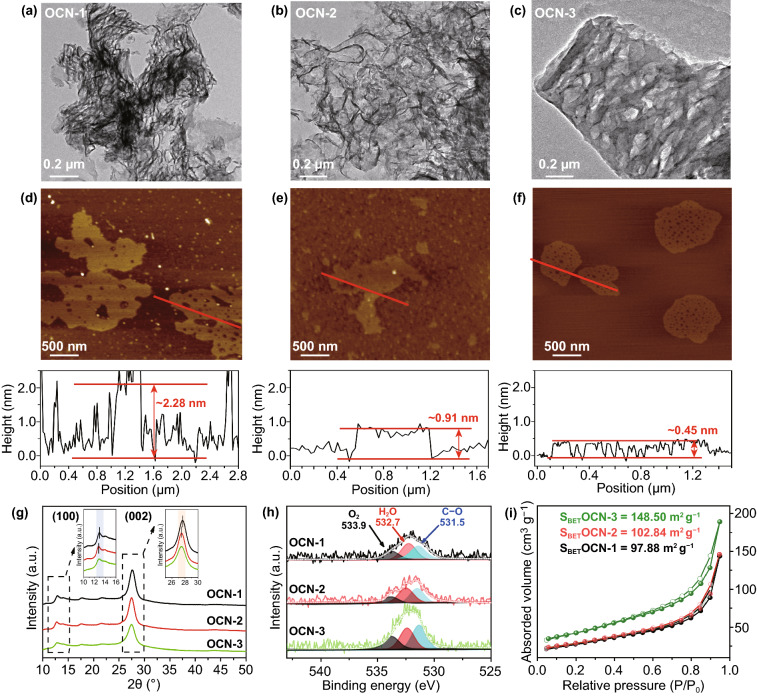
**a**–**f** TEM, AFM images and corresponding height profiles of sample. **g–i** X-ray diffraction patterns, O 1 s XPS spectra and nitrogen adsorption/desorption isotherms of OCN materials

The morphologies of a series of OCN were then analyzed by TEM and AFM. The TEM (Fig. 2a) and AFM (Fig. 2d) images displayed the typical sheet-like OCN-1 sheet was measured as 2.28 nm based on the corresponding height profiles of AFM results, indicating the OCN-1 contained 6–7 layers according to the theoretical interlayer distance of g-C_3_N_4_ (~ 0.35 nm) [36–39]. After the second thermal treatment, the size and thickness of g-C_3_N_4_ sheet (OCN-2) were decreased to ~ 1 μm and ~ 0.91 nm, respectively (Fig. 2b, e), indicating that repeating thermal treatment was helpful to obtain thinner g-C_3_N_4_ nanosheets. Further repeating thermal treatment, nearly monolayered OCN-3 nanosheet with 500 nm size and 0.45 nm thickness was obtained (Fig. 2c, f). Moreover, the pores with a uniform size of 25 nm were found on the OCN-3 nanosheets. However, the OCN-4 that was prepared by four-time thermal treatment showed a significantly decreased size (~ 160 nm), an increased thickness (~ 2.49 nm) and disappeared pores compared with those of OCN-3 (Fig. S2). As four-time thermal treatments, the obtained g-C_3_N_4_ with smaller size tended to aggregate to form the thicker g-C_3_N_4_ sheets due to the high surface energy [40]. Thus, the g-C_3_N_4_ sheets were torn into smaller fragments with the defects by the multiple thermal treatments and the three-time thermal treatment was the optimized condition for the synthesis of OCN with hollow and monolayered structures. Convinced by DFT results, the introduction of O can generate defects by distorting, then form g-C_3_N_4_ sheets with pores.

As shown in XRD patterns (Fig. 2g), two peaks at 13.0° and 27.3° could be detected, which were attributed to the in-plane repeating unites and interlayer-structure stacking of (100) and (002) of g-C_3_N_4_, respectively [41, 42]. There were no obvious changes among these OCNs, implying that the multiple thermal treatments did not affect the g-C_3_N_4_ phase composition. More importantly, these peaks tended to slightly shift toward a smaller angle, which proved again that the multiple thermal treatments were useful for expanding the interlayer space to obtain the exfoliated g-C_3_N_4_ sheets with few layers [43]. In the FT-IR spectra of products (Fig. S3a), the sharp band at ~ 810 cm^−1^ was the characteristic breathing mode of s-triazine ring. The bands from ~ 1800 to 900 cm^−1^ were the typical stretching vibration modes of C = N. The broad band between ~ 3000 and 3600 cm^−1^ was attributed to C–N heterocycles. The FT-IR results were highly consistent with g-C_3_N_4_ reported in the literature [44]. Importantly, the C–O vibration band could be clearly found at 1090 cm^−1^ and its intensity was increasing with the repeating thermal treatment, suggesting that O-containing group was successfully formed in the product. Furthermore, no N–O band (980 cm^−1^) was found, indicating that the N atoms of g-C_3_N_4_ nanosheets were not bonded with O atoms during the thermal treatment which can be evidenced by the oxygen atom site (C_2_, N_3_) from DFT computational structure. XPS survey spectra (Fig. S3b) showed that all products were composited by C, N, and O elements. The high-resolution C 1 s spectra exhibited one prominent peak in each product at 287.8 eV (Fig. S3c), which was identified as sp^2^-bonded C of N = C–(N)_2_. The high-resolution N 1 s spectra displayed three peaks at ca. 398.4, 399.9, and 400.9 eV corresponding to C = N–C, N(C)_3_, and C–N–H groups (Fig. S3d). The XPS results proved again that the typical g-C_3_N_4_ structure could be maintained during the multiple thermal treatments [37]. The C–O bond, adsorbed H_2_O, and adsorbed O_2_ could be found in high-resolution O 1 s (Fig. 2h) XPS spectra of OCN products [45]. After the multiple thermal treatments, the O content was increased from 0.84 at% for OCN-1 to 2.07 at% for OCN-4 (Table S2). According to the proportions of different kinds of O bonds in Table S3, more C–O bonds might be formed in the OCN nanosheet with the increasing thermal treatments. In addition, the change in O content was also reflected from the color of the products that were changed from yellow to light khaki with the increase in O content (Fig. S4). Overall, the current data can prove that it is a promising strategy by using a multiple thermal treatment method for the preparation of OCN with controllable O content.

The porous structure was studied by N_2_ adsorption/desorption measurement. As shown in Fig. S5, all the products displayed similar isotherm curves with a typical IV hysteresis loop. The corresponding pore-size distributions were calculated using the Barrett–Joyner–Halenda (BJH) method (Fig. S6), which showed that the products contained a broad pore-size distribution from mesopores to macropores. The OCN-3 exhibited the highest surface area of 148.50 m^2^ g^−1^ in Fig. 2i, compared with that of OCN-1 (97.88 m^2^ g^−1^), OCN-2 (102.84 m^2^ g^−1^), OCN-4 (111.92 m^2^ g^−1^). Compared with pure bulk g-C_3_N_4_ (MCN, 23.84 m^2^ g^−1^) in Fig. S7, a highly porous structure of OCN-3 monolayer structure with one atomic thickness (0.45 nm) could be beneficial to increase the surface area with abundant active sites for photocatalytic reaction [37, 45, 46].

**Fig. 3:**
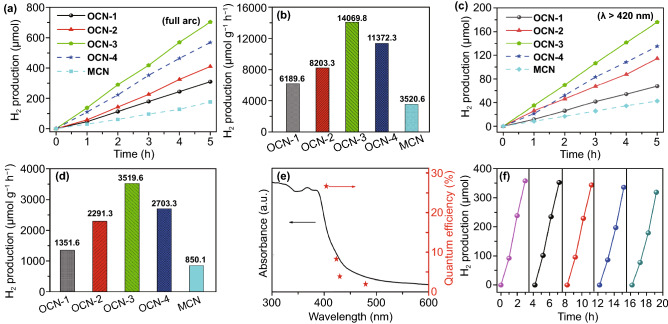
**a**–**d** Time course of hydrogen evolution and comparison of hydrogen evolution rates over 5 h for different simples under a 300 W Xe lamp without an optical filter (full arc, **a**, **b**) and with a optical filter (*λ* > 420 nm, **c**, **d**), **e–f** QE against light wavelength and cyclic photocatalytic H_2_ evolution curve of OCN-3

The photocatalytic hydrogen evolution activities of different samples were investigated. After 5 h full arc light irradiation (Fig. 3a), the total amount of produced H_2_ for OCN-1, OCN-2, OCN-3, and OCN-4 materials was 309.5, 410.2, 703.5, and 568.5 μmol, respectively. The photocatalytic hydrogen evolution activity of OCN catalysts was enhanced with increasing thermal treatment times up to three times. It decreased when the further thermal-treatment process was performed (OCN-4) due to the morphology damages and nanosheet aggregates. Among all catalysts, OCN-3 exhibited the best activity toward hydrogen evolution including the highest hydrogen produced rate of 14,069.8 μmol g^−1^ h^−1^ (Fig. 3b), which was much higher than that of OCN-1 (6189.6 μmol g^−1^ h^−1^), OCN-2 (8203.3 μmol g^−1^ h^−1^), OCN-4 (11,372.3 μmol g^−1^ h^−1^), and MCN (3520.6 μmol g^−1^ h^−1^). Even under the visible-light irradiation (*λ* ≥ 420 nm, Fig. 3c), the hydrogen evolution activity of the OCN-3 sample was also most active, and the total amount of produced H_2_ could reach 180 μmol for 5 h. The calculated hydrogen evolution rate for OCN-3 was up to 3519.6 μmol g^−1^ h^−1^ under the visible light, which was about 2.60, 1.53, 1.30, and 4.14 times higher than that of OCN-1 (1351.6 μmol g^−1^ h^−1^), OCN-2 (2291.3 μmol g^−1^ h^−1^), OCN-4 (2703.3 μmol g^−1^ h^−1^), and MCN (850.1 μmol g^−1^ h^−1^) (Fig. 3d).

To further obtain the reaction proceeds of OCN-3 through light absorption, we explored the relationship between the QE of hydrogen evolution and wavelength of incident light. It can be seen that the QE decreased with increasing wavelengths which matched well with the height variation of absorption peaks in the optical spectra. Therefore, the OCN-3 catalyst had a peak external QE of 26.96% at 400 nm and 4.28% at 420 nm (Fig. 3e), which outperformed in the g-C_3_N_4_-based catalysts reported in the literature (Table S4), including NiMo/g-C_3_N_4_, PDA@g-C_3_N_4_, Ni(OH)_2_/CdS/g-C_3_N_4_ [9, 13, 29, 39, 46]. The stability of the OCN-3 was tested by cyclic photocatalytic H_2_ evolution experiments. After five cycles, OCN-3 still exhibited the high photocatalytic activity toward hydrogen evolution reaction and only 10.4% activity was lost after 20 h (Fig. 3f). Structural information of OCN-3 after reaction was also assessed via SEM (Fig. S8) and XRD (Fig. S9) technique. Preserved morphology and similar diffraction patterns before and after cyclic test demonstrate the chemical and photophysical stability of catalysts.

**Fig. 4:**
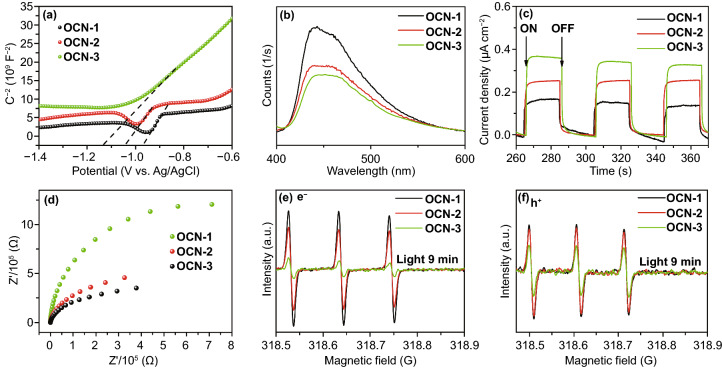
**a** Mott–Schottky plots spectra, **b** steady-state photoluminescence (PL) spectra, **c** transient photocurrent responses, **d** EIS spectroscopies, **e, f** ESR spectra of e^−^ and h^+^ of OCN-1, OCN-2, and OCN-3 under visible-light irradiation for 9 min

In order to explore the function of introducing oxygen atom toward the photophysical property of OCN, Mott–Schottky plots spectra, PL spectra were first tested. The Mott–Schottky plot was utilized to determine flat band potentials of material. All samples exhibit similar linear plots, corresponding to the character of typical n-type semiconductor in Fig. 4a [47–49]. More importantly, the derived flat potentials of OCN-3 are more negative than that of OCN-1, OCN-2, promising the enhanced reduction ability of photogenerated electrons. In Fig. 4b, all the samples exhibited a strong PL peak at ~ 445 nm, in which OCN-3 has the lowest intensity, indicated the electron/hole separation and electron transport were more efficient through OCN-3 compared with other catalysts [50]. As shown in Fig. 4c, the OCN-3 exhibited a higher photocurrent value of 0.35 μA cm^−2^ than that of OCN-1 (0.14 μA cm^−2^) and OCN-2 (0.26 μA cm^−2^), demonstrating the improvement in photocurrent response of open circuit voltage with the increasing repeat times of thermal treatment. The higher photocurrent revealed that OCN-3 has a better visible-light response and more efficient photoexcited charge separation, which was proven again by PL results. Correspondingly, the EIS Nyquist impedance spectra (Fig. 4d) showed that OCN-3 has the smallest electron-transfer resistance. The photoexcited radicals such as electrons and holes from photocatalysts were investigated by the ESR spin-trap technique with TEMPO in Fig. 4e, f. TEMPO with e^−^ or h^+^ can produce an ESR silent molecule and lead to the decrease in the intensity of TEMPO signals [51]. Hence, OCN-3 with weak signals has a large number of e^−^ and h^+^ than those of OCN-1 and OCN-2. In addition, radicals’ signal can be found in OCN-3 solution under visible-light irradiation (Fig. S10), decreasing with longer irradiated time. Therefore, the efficient separation of e^−^/h^+^, the high resistance of recombination of e^−^/h^+^, the excellent light harvest make OCN-3 active in photocatalytic hydrogen evolution reaction.

**Fig. 5:**
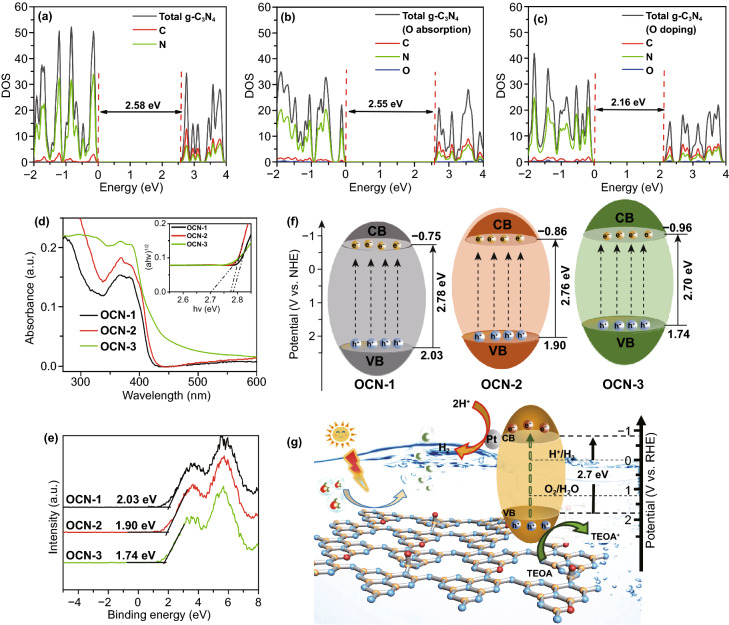
**a** Density of state (DOS) of pristine g-C_3_N_4_, **b** O-doped g-C_3_N_4_, and **c** O-adsorbed g-C_3_N_4_. **d** UV–visible absorbance spectra, **e** VB-XPS survey, and **f** energy level diagrams, **g** photocatalytic H_2_-production mechanism of OCN-1, OCN-2, OCN-3

In order to explore the function of introducing oxygen atom, HSE calculation was firstly used to obtain the theoretical density of states (DOS). According to the results of DOS, the band gap of pure g-C_3_N_4_ was about 2.58 eV in Fig. 5a which is consistent with reported results [52]. After calculating projected DOS in Fig. S11, the O-adsorption structure only caused a weak effect on band structure from pure g-C_3_N_4_ 2.58 eV to 2.55 eV (Fig. 5b). The band gap of O-doping g-C_3_N_4_ decreased from 2.58 to 2.16 eV (Fig. 5c). Such a smaller band gap is befitting for a photocatalyst. Thus, the OCN was more active than pure g-C_3_N_4_ for splitting water under light irradiation. Beside theoretical calculation, to convince the change in band structure experimentally, UV–vis DRS, VB-XPS were then studied. The UV–vis DRS result exhibited a remarkable red shift and intensity increase in absorption peak of OCN with the multiple thermal treatments (Fig. 5d), indicated the increasing harvest of visible light [53]. The band gaps of OCNs were calculated according to the Kubelka–Munk transformation, which was 2.78, 2.76, and 2.70 eV for OCN-1, OCN-2, and OCN-3, respectively. According to the VB-XPS results, the maximum of the valence band (VB) was located at 2.03, 1.90, and 1.74 eV for OCN-1, OCN-2, and OCN-3, respectively (Fig. 5e). Thus, the conduction band (CB) was determined to be − 0.97, − 1.04, and − 1.13 V (vs. Ag/AgCl, pH = 7) for OCN-1, OCN-2, OCN-3, respectively, which were converted to − 0.75, − 0.86, and − 0.96 V versus normal hydrogen electrode (vs. NHE), respectively [49]. The energy band structure can be simulated in Fig. 5f. Indeed, the CB potential of OCN-3 was smaller than that of other samples and was more negative compared with the H^+^/H_2_ reduction potential (NHE). Thus, the up-shift of conduction band energy can be resulted in a stronger reducing activity of OCN-3, leading to a significant improvement in hydrogen evolution performance. According to the above results, we considered that O-doping could lead g-C_3_N_4_ to a significant change in band gap. This function could be ignored for O-adsorption. However, the O-adsorption still played an important role in the generation of defects that were contributed to the formation of hollow morphology.

A possible photocatalytic hydrogen production mechanism was proposed, as shown in Fig. 5g. The e^−^ and h^+^ pairs were generated when the OCN was irradiated by the visible light. Then, e^−^ in CB transferred to Pt for releasing H_2_ by reducing H_2_O, while the generated h^+^ in VB reacted with TEOA to inhibit the recombination of e^−^/h^+^. Since the above steps were intimately associated with the intrinsic characteristics of photocatalyst, such as electronic, surface, and textural structures, an integrated engineering of the above properties would enable a synergetic tuning and optimization to further improve the photocatalytic performance. In our work, we modified the morphology and electronic structure of g-C_3_N_4_ by introducing O using the multiple thermal treatments. On the one hand, the O-adsorption can create more defects in g-C_3_N_4_ nanosheets, leading to the formation of a hollow structure that was contributed to the improved surface area. On the other hand, the O-doping can reduce the band gap of g-C_3_N_4_. The separation of e^−^/h^+^ was promoted through g-C_3_N_4_, while the recombination of the generated e^−^/h^+^ was inhibited due to the fast transport of electrons over g-C_3_N_4_. In doing so, the OCN showed an excellent photocatalytic performance and it holds a promising application such as photocatalysts for hydrogen evolution and substrates for the synthesis of catalysts composites.

## Conclusion

In summary, we demonstrated a novel approach to synthesize OCN with structure regulation and morphology control by using the multiple thermal treatments under the N_2_/O_2_ atmosphere. The physical characterizations and theorized calculations proved that the multiple thermal treatments played a crucial role in morphology control and structure regulation by introducing O atoms. There were two kinds of O-incorporated structures. One was O-adsorption that could create a lot of defects to the formation of hollow and monolayered structure. Another was O-doping which can reduce the band gap significantly. Owing to this variation in structure, the optimized OCN-3 showed an excellent visible-light photocatalytic activity toward hydrogen production. The hydrogen evolution activity of OCN-3 was 3519.6 μmol g^−1^ h^−1^ for ~ 20 h, which is over four times higher than that of pure bulk g-C_3_N_4_ (850.1 μmol g^−1^ h^−1^). Besides, OCN-3 exhibited a stable photocatalytic activity due to oxygen functions which only 10.4% activity was lost after 20 h. This work not only demonstrated a powerful strategy to synthesize porous and ultrathin g-C_3_N_4_ nanosheet with highly efficient photocatalytic H_2_ evolution by the function of oxygen, but also paves a new avenue to optimize the electronic, surface, and textural structure for excellent photocatalysts.

## Supporting information

Below is the link to the electronic supplementary material. Supplementary material 1 (PDF 5077 kb)
