# Supplementary material for: Band Engineering and Morphology Control of Oxygen-Incorporated Graphitic Carbon Nitride Porous Nanosheets for Highly Efficient Photocatalytic Hydrogen Evolution
Source: Nanomicro Lett. 2021 Jan 4;13:48. doi: 10.1007/s40820-020-00571-6 (PMC8187501; doi:10.1007/s40820-020-00571-6)
Supplement: Supplementary file 1 — Supplementary material 1 (PDF 5077 kb) [file 40820_2020_571_MOESM1_ESM.pdf]

Supporting Information for

Band Engineering and Morphology Control of Oxygen-incorporated Graphitic Carbon Nitride Porous Nanosheets for Highly Efficient Photocatalytic Hydrogen Evolution

Yunyan Wu[1, 2, #], Pan Xiong[1, #], Jianchun Wu[2, 3], Zengliang Huang[2], Jingwen Sun[1], Qinqin Liu[2], Xiaonong Cheng[2], Juan Yang[1, 2, *], Junwu Zhu[1, *], Yazhou Zhou[2, *]

[1]Key Laboratory for Soft Chemistry and Functional Materials, Ministry of Education, Nanjing University of Science and Technology, Nanjing 210094, China

[2]School of Materials Science and Engineering, Jiangsu University, Zhenjiang 212013, China

[3]Institute of Nuclear Science and Technology, Sichuan University, Chengdu 610064, China

[#]Yunyan Wu and Pan Xiong have contributed equally to this work

*Corresponding authors. E-mail: yangjuan6347@mail.ujs.edu.cn (J. Yang); zhujw@njust.edu.cn (J. Zhu); yazhou@ujs.edu.cn (Y. Zhou)

S1 Quantum Efficiency Calculations

The apparent quantum yield (AQY) for hydrogen evolution was measured under monochromatic light irradiation (400, 420, 435, and 450 nm) using a 300W Xenon lamp with specific band-pass filters. Depending on the amount of hydrogen produced in one hour, the AQY was calculated from Eqs. (S1)-(S4):

$$\text{AQY} = \frac{\text{Number of evolved hydrogen molecules} \times 2}{\text{Number of incident photons}} \times 100\% \quad \text{(S1)}$$

$$\text{Number of evolved hydrogen molecules} = 2 \times M \times N_A \quad \text{(S2)}$$

$$\text{Number of incident photons} = \frac{E \times \lambda}{h \times c} \times 100\% \quad \text{(S3)}$$

$$\text{AQY} = \frac{2 \times M \times N_A \times h \times c}{S \times P \times \lambda \times t} \times 100\% \quad \text{(S4)}$$

Where, M is the mole number of hydrogen molecules (mol), N_A is Avogadro constant (6.022×10^{23} mol^{-1}), h is Plank constant (6.626×10^{-34} J S), c is the speed of light (3×10^{8} m s^{-1}), λ is the monochromatic light wavelength (m), P is the average intensity of irradiation, S is the irradiation area (cm^2), and t is the photoreaction time (s).

Table S1 Calculated apparent quantum efficiency (AQE) at different wavelengths

Wavelength	H_2 Evolved (μmol)	Light Intensity	AQE
λ=400nm	60.95	12.53 mW	26.96%
λ=420nm	10. 88	13.41 mW	4.28%
λ=435nm	4.42	14.55 mW	1.55%
λ=450nm	0	15.12 mW	0%

$AQE(\%)=\frac{the\ number\ of\ reacted\ electrons}{the\ number\ of\ incident\ photos}\times 100\%=\frac{2\times the\ number\ of\ evolved\ H_2\ molecules}{N}\times 100\%=$

$=\frac{2N_A C}{N}$ $\quad (N=\frac{E\lambda}{hc}=\frac{Pt\lambda}{hc})$

C: H_2 production amount *N_A: Avogadro constant*

P: Light intensity *t: Photocatalytic reaction time*

λ: The wavelength of light *h: Planck constant*

c: The speed of light

(a) λ=400 nm

$N=\frac{E\lambda}{hc}=\frac{12.53\times 10^{-3}\times 3\times 3600\times 400\times 10^{-9}}{6.626\times 10^{-34}\times 3\times 10^{8}}=2.722\times 10^{20}$

$AQE=\frac{the\ number\ of\ reacted\ electrons}{the\ number\ of\ incident\ photos}\times 100\% = \frac{2\times the\ number\ of\ evolved\ H_2\ molecules}{N}\times 100\%=$
$\frac{2\times 6.02\times 10^{23}\times 60.95\times 10^{-6}}{2.722\times 10^{20}}=26.96\%$

(b) λ=420 nm

$N=\frac{E\lambda}{hc}=\frac{13.41\times 10^{-3}\times 3\times 3600\times 420\times 10^{-9}}{6.626\times 10^{-34}\times 3\times 10^{8}}=3.0598\times 10^{20}$

$AQE=\frac{the\ number\ of\ reacted\ electrons}{the\ number\ of\ incident\ photos}\times 100\% = \frac{2\times the\ number\ of\ evolved\ H_2\ molecules}{N}\times 100\%=$
$\frac{2\times 6.02\times 10^{23}\times 10.88\times 10^{-6}}{3.0598\times 10^{20}}=4.28\%$

(c) λ=435 nm

$N=\frac{E\lambda}{hc}=\frac{14.55\times 10^{-3}\times 3\times 3600\times 435\times 10^{-9}}{6.626\times 10^{-34}\times 3\times 10^{8}}=3.438\times 10^{20}$

$AQE=\frac{the\ number\ of\ reacted\ electrons}{the\ number\ of\ incident\ photos}\times 100\% = \frac{2\times the\ number\ of\ evolved\ H_2\ molecules}{N}\times 100\%=$
$\frac{2\times 6.02\times 10^{23}\times 4.42\times 10^{-6}}{3.438\times 10^{20}}=1.55\%$

(d) λ=450 nm

$N=\frac{E\lambda}{hc}=\frac{15.12\times 10^{-3}\times 3\times 3600\times 450\times 10^{-9}}{6.626\times 10^{-34}\times 3\times 10^{8}}=3.697\times 10^{20}$

$AQE=\frac{the\ number\ of\ reacted\ electrons}{the\ number\ of\ incident\ photos}\times 100\% = \frac{2\times the\ number\ of\ evolved\ H_2\ molecules}{N}\times 100\%=$
$\frac{2\times 6.02\times 10^{23}\times 0\times 10^{-6}}{3.697\times 10^{20}}=0\%$

S2 Supplementary Tables and Figures

Table S2 XPS comparison of element contents of the different samples

Sample type	N content (at%)	O content (at%)	C content (at%)
OCN -1	60.29	0.84	38.86
OCN -2	59.88	1.02	39.10
OCN -3	59.07	1.62	39.31
OCN -4	58.04	2.07	39.88

Table S3 O bond proportions of the different samples

Sample type	O_2 content (%)	H_2O content (%)	C-O content (%)
OCN -1	23.12	47.45	29.42
OCN -2	19.62	44.66	35.71
OCN -3	15.83	42.52	41.63
OCN -4	14.63	42.46	42.91

Table S4 H_2 evolution rate of other reported CN-based photocatalysts

Composite type	Dopant /cocatalysts	Reactant solution and sacrificial agent	Light source	Activity (μmolg^{-1}h^{-1})	Refs.
NiMo/g-C_3N_4	1 wt% Pt	100 mL aqueous solution, TEOA (10 vol%)	Xe-lamp (300 W), λ > 420 nm	1785	[S1]
MS-550	3 wt% Pt	100 mL aqueous solution, TEOA (10 vol%)	Xe-lamp (300 W), λ > 420 nm	661	[S2]
T-CN	3 wt% Pt	100 mL aqueous solution, TEOA (10 vol%)	Xe-lamp (300 W), λ > 420 nm	332	[S3]
CN-Na-7	3 wt% Pt	100 mL aqueous solution, TEOA (10 vol%)	Xe-lamp (300 W), λ > 420 nm	169	[S4]
CNQ680	3 wt% Pt	300 mL aqueous solution, TEOA (10vol%)	Xe-lamp (300 W), λ > 440 nm	310	[S5]
U-CN-6	3 wt% Pt	100 mL aqueous solution, TEOA (10 vol%)	300 W Xenon lamp, λ > 420 nm	812	[S6]
g-C_3N_4 @PDA	3 wt% Pt	100 mL aqueous solution, TEOA (10 vol%)	300 W Xe-lamp, λ > 420 nm	377.7	[S7]
3DCN1	3 wt% Pt	100 mL aqueous solution, TEOA (10 vol%)	300 W Xenon lamp, λ > 420 nm	1681	[S8]
P-DCN	3 wt% Pt	100 mL aqueous solution, TEOA (10 vol%)	300W Xeon-lamp, λ > 420 nm	2092	[S9]
$CNU_{0.075}$	1 wt% Pt	100 mL aqueous solution, TEOA (10 vol%)	300W Xenon arc lamp, λ > 420 nm	1003.9	[S10]
Co(Mo-Mo_2C)/g-C_3N_4	2 wt% Pt	10 mL aqueous solution, TEOA (20 vol%)	300W Xenon arc lamp, λ > 420 nm	4291	[S11]
OCN	3 wt% Pt	100 mL aqueous solution, TEOA (10 vol%)	300W Xenon arc lamp, λ > 420 nm	3519.6	Our Work

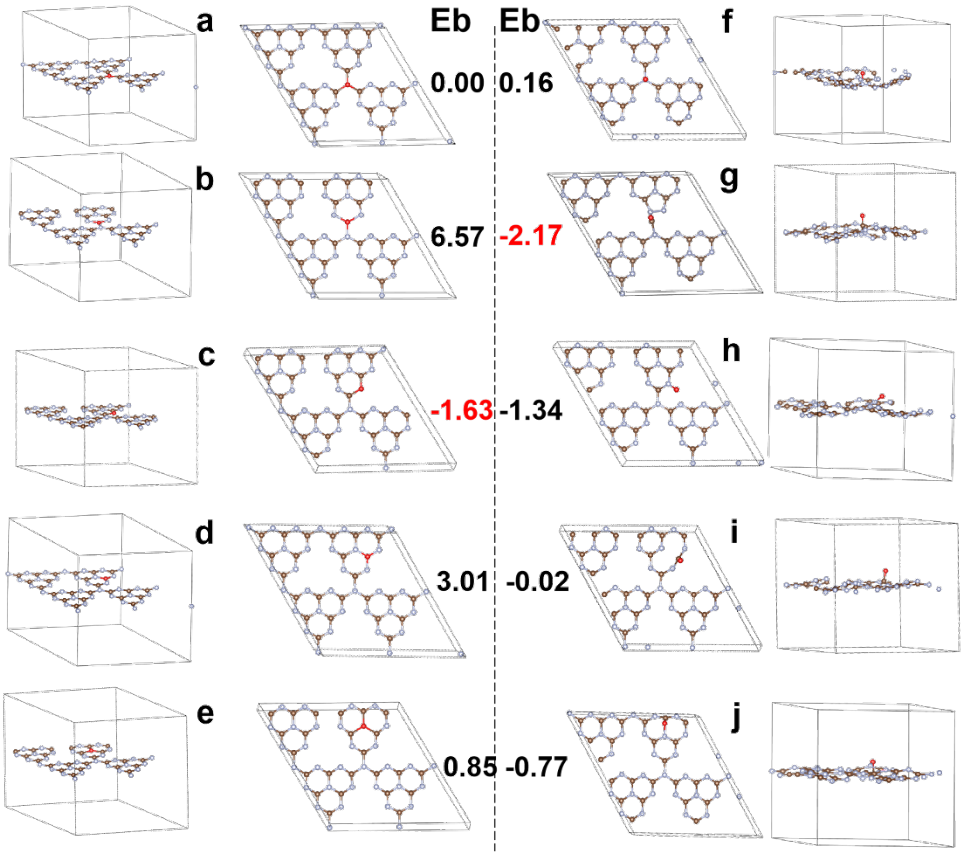

Fig. S1 a-e Optimized atomic structure view and energy of O-doped g-C_3N_4 and **f-j** O-adsorbed g-C_3N_4

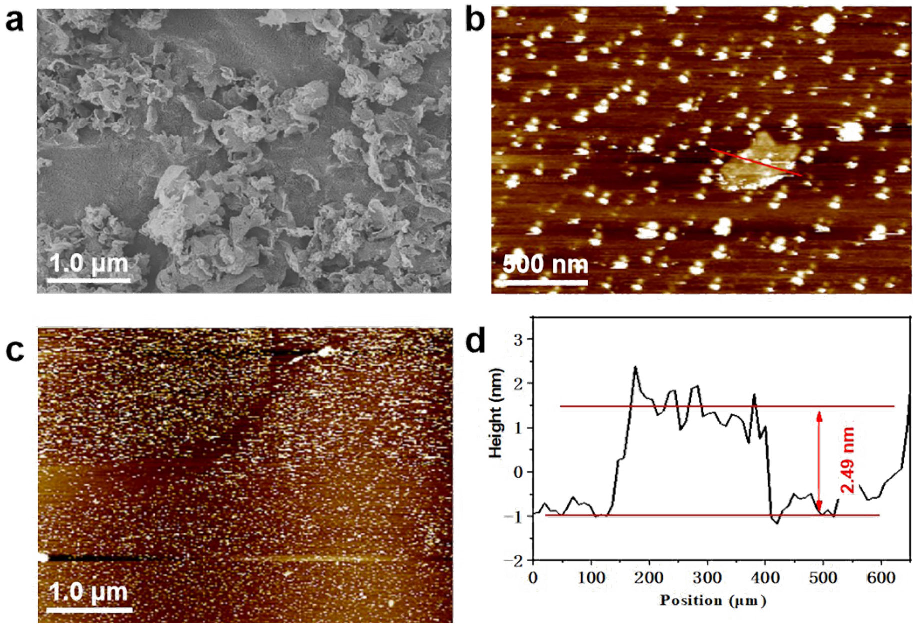

Fig. S2 a SEM, **b-d** AFM images of OCN-4

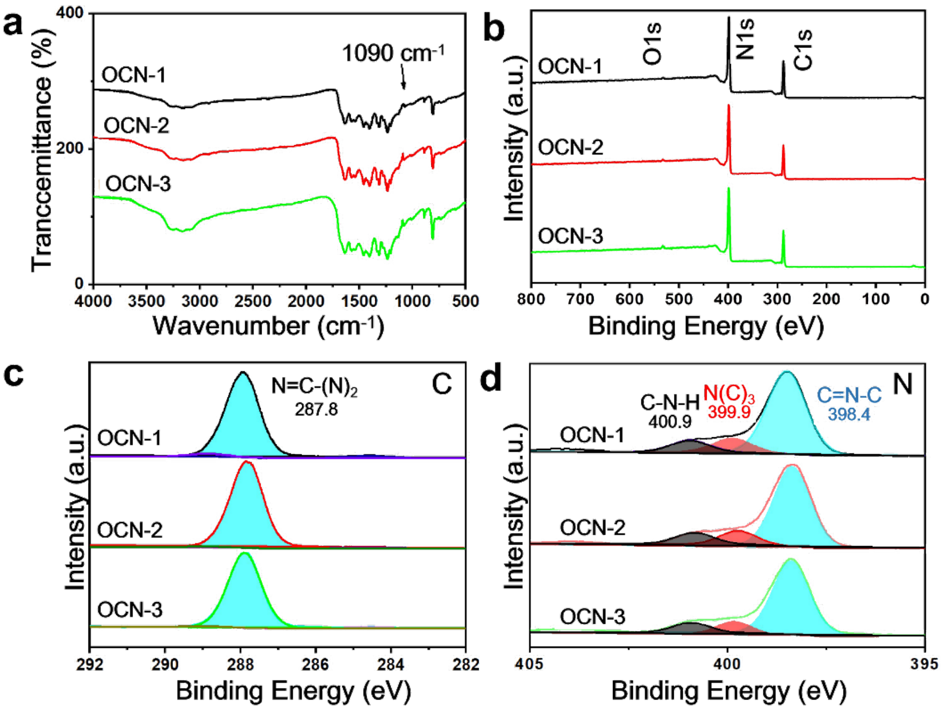

Fig. S3 **a** FT-IR, **b** survey, **c-d** C1s and N1s XPS spectra of OCN

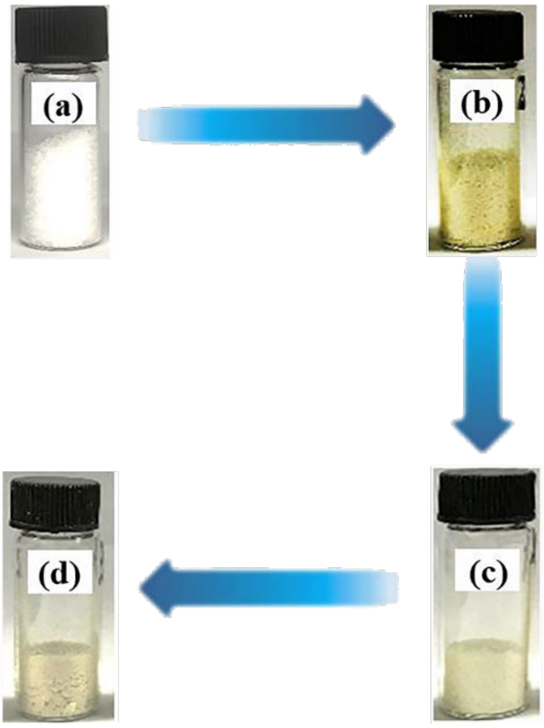

Fig. S4 **a-d** Digital photographs of powders: **a** precursor urea, **b** OCN-1, **c** OCN-2, **d** OCN-3

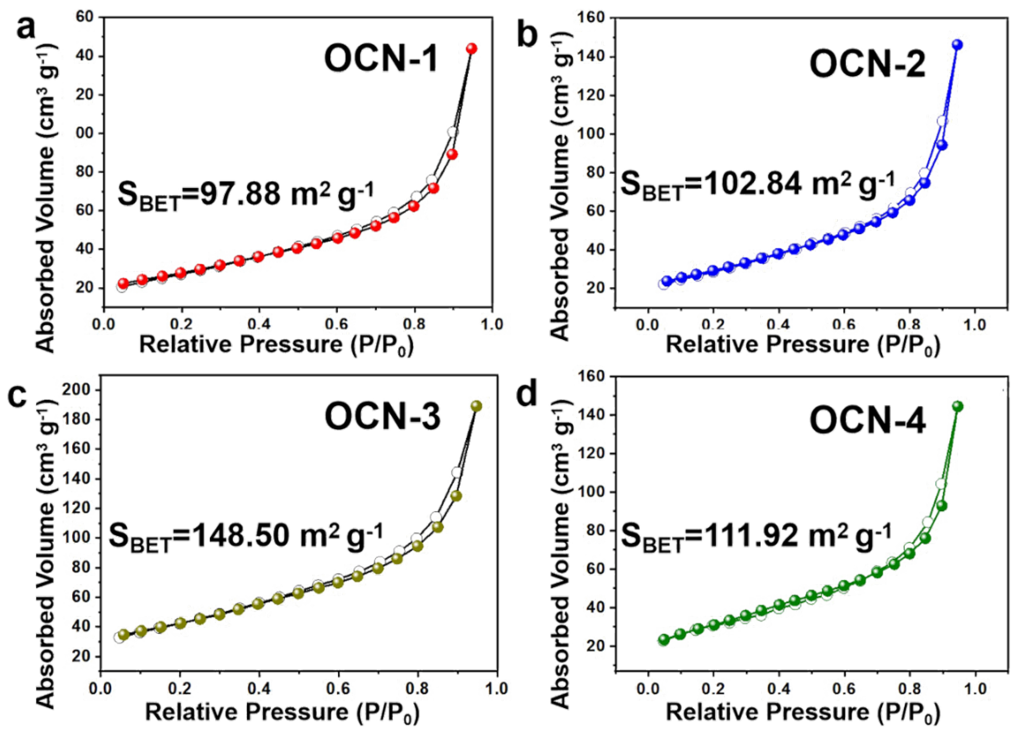

Fig. S5 a-d Nitrogen adsorption/desorption isotherms of **a** OCN-1, **b** OCN-2, **c** OCN-3, **d** OCN-4

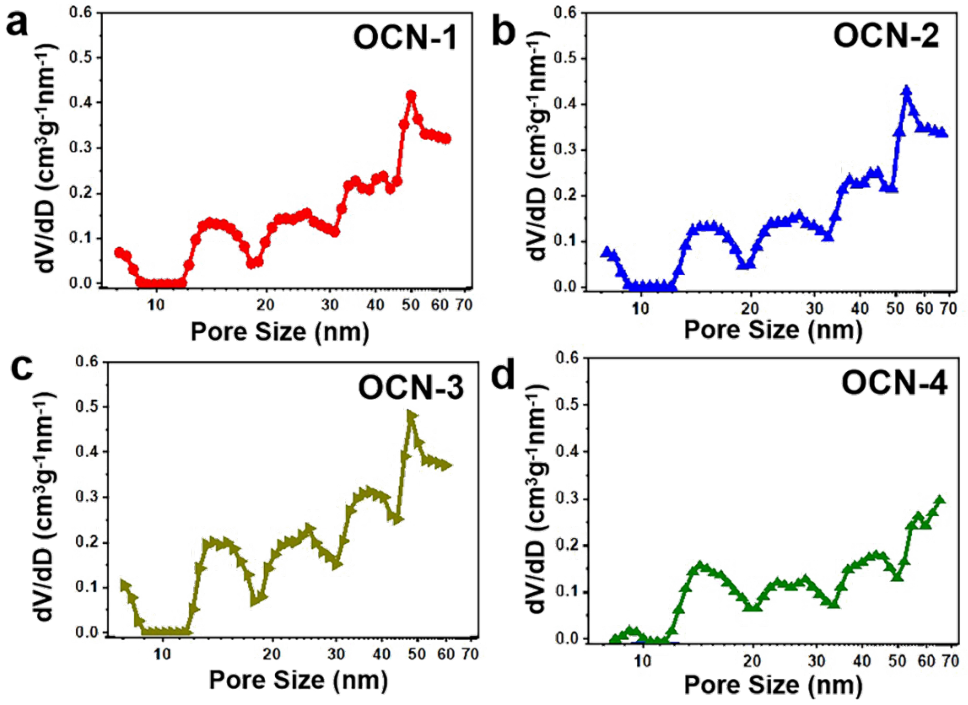

Fig. S6 a-d Pore size distribution curves of **a** OCN-1, **b** OCN-2, **c** OCN-3, **d** OCN-4

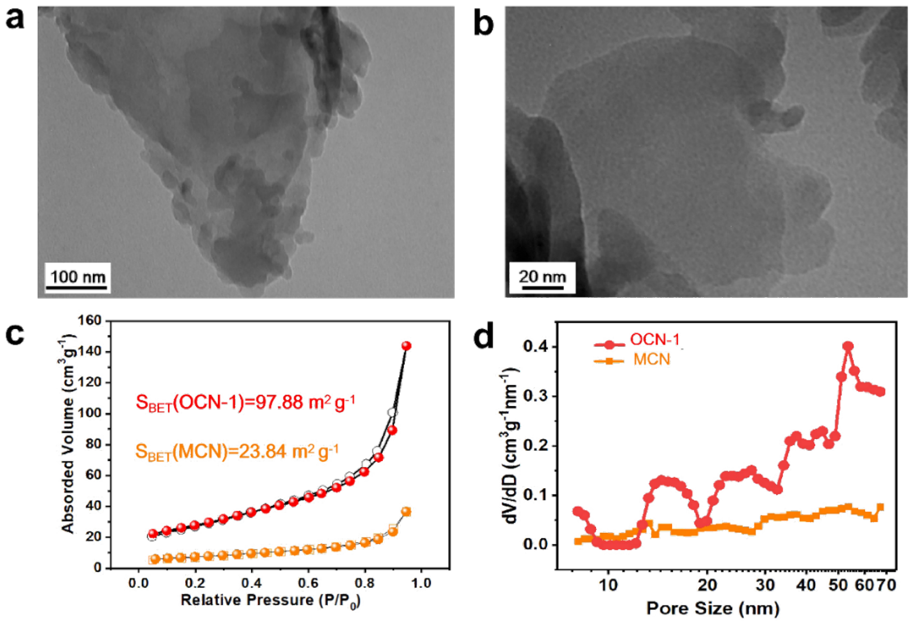

Fig. S7 a-b TEM images of MCN, **c** nitrogen adsorption/desorption isotherms and **d** pore size distribution curves of OCN-1, MCN

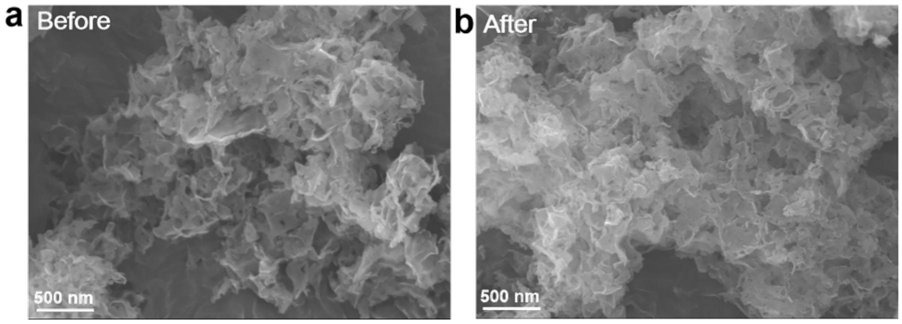

Fig. S8 a-b SEM images of OCN-3 before and after cyclic photocatalytic H_2 evolution tests

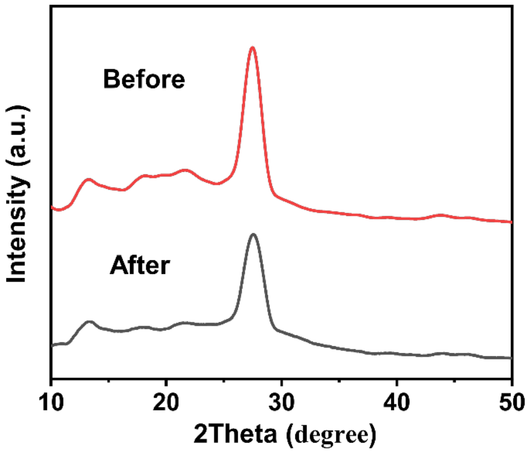

Fig. S9 XRD patterns of OCN-3 before and after cyclic photocatalytic H_2 evolution tests

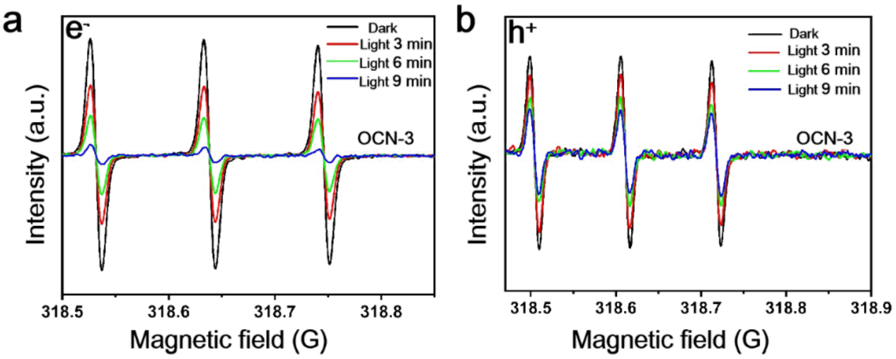

Fig. S10 a-b ESR spectra of e^- and h^+ for various times of OCN-3 under visible light irradiation

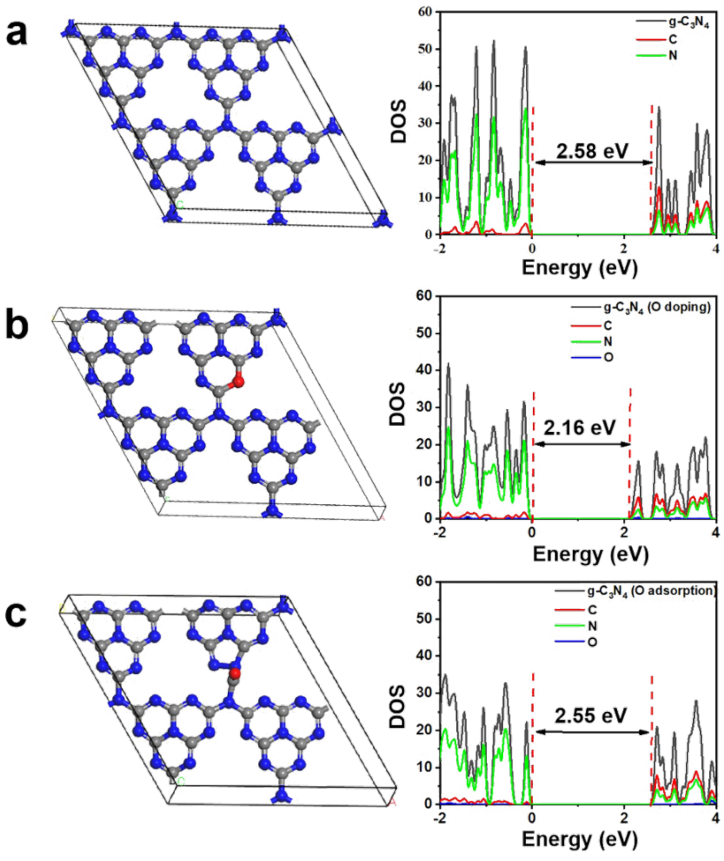

Fig. S11 a-c Density of state (DOS) of **a** pristine g-C_3N_4, **b** O-doped g-C_3N_4 and **c** O-adsorbed g-C_3N_4

Supplementary References

[S1] X. Han, D. Xu, L. An, C. Hou, Y. Li et al., Ni-Mo nanoparticles as co-catalyst for drastically enhanced photocatalytic hydrogen production activity over g-C_3N_4. Appl. Catal. B: Environ. **243**, 136-144 (2019). https://doi.org/10.1016/j.apcatb.2018.10.003

[S2] J. Yang, Y. Liang, K. Li, G. Yang, K. Wang et al., One-step synthesis of novel K^+ and cyano groups decorated triazine-/heptazine-based g-C_3N_4 tubular homojunctions for boosting photocatalytic H_2 evolution. Appl. Catal. B: Environ. **262**, 118252 (2020). https://doi.org/10.1016/j.apcatb.2019.118252

[S3] J. Yang, Y. Liang, K. Li, G. Yang, K. Wang et al., Cyano and potassium-rich g-C_3N_4 hollow tubes for efficient visible-light-driven hydrogen evolution. Catal. Sci. Technol. **9**, 3342-3346 (2019). https://doi.org/10.1039/C9CY00925F

[S4] Y. Shang, Y. Ma, X. Chen, X. Xiong, J. Pan, Effect of sodium doping on the structure and enhanced photocatalytic hydrogen evolution performance of graphitic carbon nitride. Molecular Catal. **433**, 128-135 (2017). https://doi.org/10.1016/j.mcat.2016.12.021

[S5] P. Niu, M. Qiao, Y. Li, L. Huang, T. Zhai, Distinctive defects engineering in graphitic carbon nitride for greatly extended visible light photocatalytic hydrogen evolution. Nano Energy **44**, 73-81 (2018). https://doi.org/10.1016/j.nanoen.2017.11.059

[S6] C. Dong, Z. Ma, R. Qie, X. Guo, C. Li et al., Morphology and defects regulation of carbon nitride by hydrochloric acid to boost visible light absorption and photocatalytic activity. Appl. Catal. B: Environ. **217**, 629-636 (2017). https://doi.org/10.1016/j.apcatb.2017.06.028

[S7] H. Wang, Q. Lin, L. Yin, Y. Yang, Y. Qiu et al., Biomimetic design of hollow flower-like g-C_3N_4@PDA organic framework nanospheres for realizing an efficient photoreactivity. Small **15**, 1900011 (2019). https://doi.org/10.1002/smll.201900011

[S8] J. Sun, F. Yao, L. Dai, J. Deng, H. Zhao et al., Task-specific synthesis of 3D porous carbon nitrides from the cycloaddition reaction and sequential self-assembly strategy toward photocatalytic hydrogen evolution. ACS Appl. Mater. Inter. **12**, 40433-40442 (2020). https://doi.org/10.1021/acsami.0c14097

[S9] D. Zhang, Y. Guo, Z. Zhao, Porous defect-modified graphitic carbon nitride via a facile one-step approach with significantly enhanced photocatalytic hydrogen evolution under visible light irradiation. Appl. Catal. B: Environ. **226**, 1-9 (2018). https://doi.org/10.1016/j.apcatb.2017.12.044

[S10] Q. Xu, B. Zhu, B. Cheng, J. Yu, M. Zhou et al., Photocatalytic H_2 evolution on graphdiyne/g-C_3N_4 hybrid nanocomposites. Appl. Catal. B: Environl. **255**, 117770 (2019). https://doi.org/10.1016/j.apcatb.2019.117770

[S11] Y. Zheng, J. Dong, C. Huang, L. Xia, Q. Wu et al., Co-doped Mo-Mo_2C cocatalyst for enhanced g-C_3N_4 photocatalytic H_2 evolution. Appl. Catal. B: Environl. **260**, 118220 (2020). https://doi.org/10.1016/j.apcatb.2019.118220
